# MOF-808 as an Efficient Catalyst for Valorization of Biodiesel Waste Production: Glycerol Acetalization

**DOI:** 10.3390/ma16217023

**Published:** 2023-11-03

**Authors:** Fátima Mirante, Pedro Leo, Catarina N. Dias, Luís Cunha-Silva, Salete S. Balula

**Affiliations:** 1 LAQV/REQUIMTE, Department of Chemistry and Biochemistry, Faculty of Sciences, University of Porto, 4169-007 Porto, Portugal; fatimaisabelmirante@gmail.com (F.M.); pedro.leo@urjc.es (P.L.); up201804900@edu.fc.up.pt (C.N.D.); 2 Department of Chemical and Environmental Technology, Universidad Rey Juan Carlos, Calle Tulipán s/n, 28933 Móstoles, Spain

**Keywords:** glycerol, acetalization, solketal, catalysis, MOF-808

## Abstract

Glycerol is the main residue in the biodiesel production industry; therefore, their valorization is crucial. The acetalization of glycerol toward fuel additives such as solketal (2,2-dimethyl-1,3-dioxolan-4-methanol) is of high interest, promoting circular economy since it can be added to biodiesel or even fossil diesel to improve their quality and efficiency. Straightforward-prepared metal–organic framework (MOF) materials of the MOF-808 family were applied to the valorization of glycerol for the first time. In particular, MOF-808(Hf) was revealed to be an effective heterogeneous catalyst to produce solketal under moderate conditions: a small amount of the MOF material (only 4 wt% of glycerol), a 1:6 ratio of glycerol/acetone, and a temperature of 333 K. The high efficiency of MOF-808(Hf) was associated with the high amount of acid centers present in its structure. Furthermore, its structural characteristics, such as window opening cavity size and pore diameters, were shown to be ideal for reusing this material for at least ten consecutive reaction cycles without losing activity (conversion > 90% and selectivity > 98%). Remarkably, it was not necessary to wash or activate the MOF-808(Hf) catalyst between cycles (no pore blockage occurred), and it maintained structural integrity after ten cycles, confirming its ability to be a sustainable heterogeneous catalyst for glycerol valorization.

## 1. Introduction

The current energy system is mainly supported by fossil fuels, which are responsible for most of the atmospheric pollutants emitted by human activity, causing serious environmental concerns [1]. Climate change and the energy crisis have boosted the use of biomass as an energy source in recent years. This mitigates climate change and, in turn, can provide an alternative energy source to increase energy security [2]. The global production of biodiesel is growing as never seen before, although it generates massive amounts of crude of glycerol as a by-product, in an amount of 10–12% from produced biodiesel and with a purity near 50–55% [3]. Various different applications of refined glycerol are reported, namely in cosmetics, pharmaceuticals, and food industries. This use of glycerol represents approximately 65.1% of the total glycerol market [4]. However, several sectors of industry are not able to use and convert crude glycerol that resulted from biodiesel production, mainly due to its low purity [5]. Among these alternatives, acetalization reaction is a process that adheres to the principles of Green Chemistry as the reagents are from renewable sources, the reaction is catalyzed by recycle and recover catalysts, the toxicity of chemicals involved is low, and water is the by-product [6,7]. This allows for the production of solketal as the main product and acetal and water as by-products. At present, solketal has a market value of around 3000 USD/ton, providing additional revenue opportunities for the biodiesel production industry and the agricultural area [8,9]. Traditionally, the condensation of glycerol with acetone has been performed using Brønsted and Lewis acids [10] such as H_2_SO_4_ [11], Amberlyst-15 and Amberlyst-36 [12,13], silica-supported heteropoly acids [14], mesoporous silicates containing aryl sulfonate groups [15], and zeolite [16]. Over the past two decades, metal−organic frameworks (MOFs), a class of porous materials built from the coordination of organic linkers and metal ions, have drawn scientific interest over other porous materials due to the possibility of tuning their structure and consequent property features, as well as an excellent porosity [17]. Due to their structural versatility, MOFs can lodge Lewis and Bronsted sites that improve their properties as heterogeneous catalysts [18,19,20]. Few works have evaluated MOF materials as heterogeneous catalysts in the acetalization reaction [21,22,23,24,25,26]. Bakuru and co-workers were able to obtain the highest catalytic performance of solketal due to the oxophilicity of the metal ions present in the UiO-66 MOFs (Zr, Ce, and Hf) [22]. In this sense, the versatility of Zr-based nodes as structural elements originates a series of MOF structures with 12-, 10-, 8-, and 6-connected nodes. It has been found in a variety of transformations, such as catalytic carbonyl transfer hydrogenation [27], epoxide ring-opening reaction [28], and hydrolysis of nerve-agent simulants, ref. [29] that MOF-808(Zr) (6-connected) exhibits higher catalytic activity compared to UiO-66 (Zr). This is due to the coordinatively unsaturated units existing in MOF-808(Zr). The nodes are not fully coordinated; thus, the terminal Zr-OH/Zr-OH_2_ face the pores in the MOF together with better textural properties, therefore allowing a much higher percentage of active nodes to act as catalysts [30].

The potential of the porous MOF-808 (Zr and Hf) materials as heterogeneous catalysts in the acetalization reaction of glycerol with acetone to produce solketal was investigated and reported following our research group’s recent interest in the application of MOF and MOF-based materials as heterogeneous catalysts in sustainable processes [31,32,33,34,35]. The influence of different reaction parameters, such as temperature, catalyst amount, and reactants ratio, was evaluated to determine the best operation conditions for the revalorization of glycerol. The reusability and the stability after catalytic use were studied along several successive catalytic runs.

## 2. Experimental Section

### 2.1. Materials and Methods

All reagents and solvents were purchased from commercial sources and used without further purification: Glycerol (99.92%, Fluka, London, UK), acetone (≥99%, Sigma-Aldrich, St. Louis, MI, USA), methanol (>99.8%, Merck, Rahway, NJ, USA), sodium chloride (>99.5%, Panreac, Chicago, IL, USA), zirconium(IV) chloride (ZrCl_4_, ≥99.5%, Aldrich, St. Louis, MI, USA), dimethylformamide (DMF, >99.8%, Merck), ethanol (CH_3_OH, ≥99.8%, Fisher Chemical, Hampton, NH, USA), H_2_BTC (1,3,5-benzenetricarboxylic acid, >95%, Aldrich, St. Louis, MI, USA), acetic acid (CH_3_COOH, Merck), hafnium (IV) chloride (HfCl_4_, Alfa Aesar, Haverhill, MA, USA), and formic acid (HCOOH, 90%, Fisher Chemical).

Powder X-ray diffraction (XRD) patterns were obtained at room temperature on a Rigaku’s Smartlab diffractometer operating with a Cu radiation source (*λ* = 1.540593 Å) and in a Bragg–Brentano θ/2θ configuration (45 kV, 200 mA). Intensity data were collected by a step-counting method (step 0.01°), in continuous mode, in the 3 ≤ 2θ ≤ 60° range, and all the representations are shown in arbitrary unities of intensity. Fourier-transformed Infrared (FTIR) spectra were acquired on the attenuated total reflectance (ATR) operation mode of a Perkin Elmer FTIR System Spectrum BX spectrometer, and all the representations are shown in arbitrary unities of transmittance. Argon adsorption–desorption isotherms at −186 °C were measured using AutoSorb equipment (Quantachrome Instruments, Boynton Beach, FL, USA). Samples were previously evacuated in situ under a high vacuum (10^−7^ bar) for 12 h at 100 °C. The surface area was calculated by using the Brunauer–Emmett–Teller (BET) model [36]. The pore volume and diameter were estimated using non-local DFT calculations, assuming a kernel model of Ar at −186 °C on carbon (cylindrical pores, NLDFT equilibrium model) [37]. Scanning electron microscope (SEM) measurements and associated EDX elemental analysis were performed on a Quanta 400 FEG ESEM electron microscope operating at 200 kV accelerating voltage. The strength of acidity of the MOF-808 materials was calculated using a potentiometric titration using 2 mol dm^−3^ NaCl as a cation-exchange agent. The two different MOF-808 (Zr and Hf) structures were maintained in contact with NaCl solution (1:1 ratio) at room temperature for 24 h under stirring. The suspension was separated by filtration. The final solution was titrated with 0.04 M NaOH solution to determine the loading of acid sites of the two MOFs-808 [38].

### 2.2. Preparations of the Materials

MOF-808(Zr) was synthesized according to published procedures with slight modifications [1,2]. Briefly, ZrOCl_4_·8H_2_O (2.17 mmol, 701 mg) and H2BTC (1 mmol, 214 mg) in a solvent mixture of DMF/CH_3_COOH (30 mL/18 mL) were placed in a beaker and stirred at room temperature for 30 min. Then, the solution was transferred into an autoclave lined with Teflon and heated at 403 K for 48 h. After cooling down to room temperature, the white precipitate was centrifuged and washed three times with DMF and ethanol. The obtained MOF was dried at 80 °C under a vacuum for 12 h.

MOF-808(Hf) was prepared according to previously reported procedures and slight modifications [39]. HfCl_4_ (5 mmol, 1.63 g) was dissolved in a mixture of H_2_O/HCOOH (30 mL/20 mL) and stirred at room temperature until we received a translucid solution. Then, H_2_BTC (5 mmol, 1.05 g) ligand was added, and the flask was placed in an oil bath with refluxed at 373 K for 12 h. The obtained white MOF powder was centrifuged and washed with water and methanol three times each solvent. The MOF powder was dried at 100 °C under vacuum for 24 h. Obtained materials were carefully characterized using several techniques to confirm their successful preparation: FTIR-ATR, powder XRD, Argon adsorption–desorption isotherms, and SEM/EDS.

### 2.3. Catalytic Studies

A typical acetalization catalytic reaction for the valorization of glycerol with acetone was performed under air in a closed borosilicate 5 mL vessel equipped with a magnetic stirrer and immersed in a thermostatically controlled liquid paraffin bath (298, 313, and 333 °C). For each run, the solution based on glycerol and acetone was preheated to the chosen temperature (25–60 °C) for 10 min, and then the catalyst (2, 4, and 8 wt%, based on the glycerol weight) was added, starting the reaction. The reaction evolution and product analysis were controlled by GC-FID analysis carried out in a Varian CP-3380 chromatograph. Hydrogen was used as carrier gas with a 30 mL min^−1^ flow rate, and a Suprawax-280 capillary column (30 m × 0.25 mm i.d.; 0.25 μm film thickness) was used. Products obtained were identified by GC-MS, using a Hewlett Packard 5890 chromatograph equipped with a Mass Selective Detector MSD series II employing He as the carrier gas (35 cm s^−1^). Catalytic experiments were repeated at least thrice, and the error was equal to or inferior to 5%.

## 3. Results and Discussion

### 3.1. Catalysts Characterization

**Figure 1:**
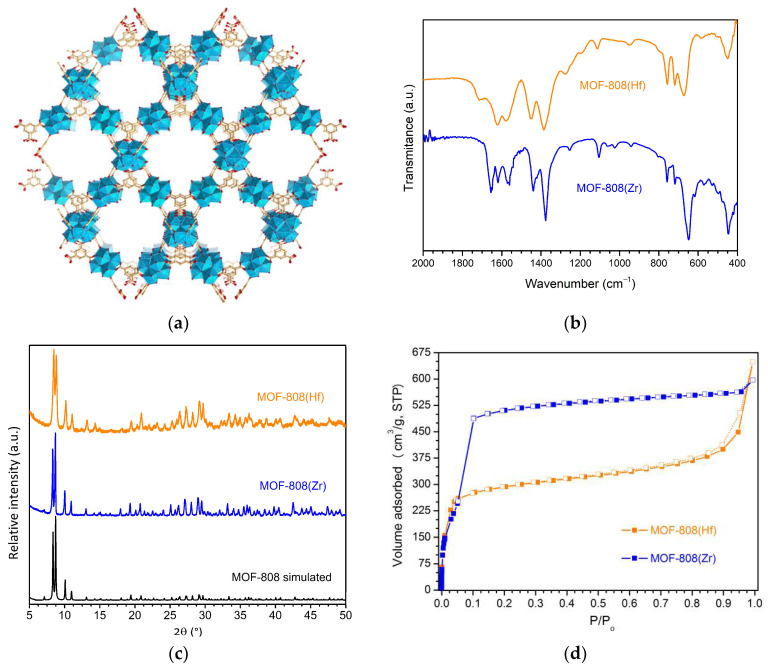

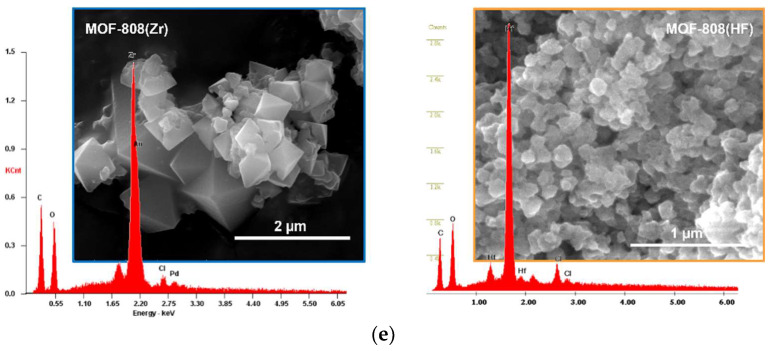
(**a**) Extended crystalline structure representation of MOF-808. (**b**) FTIR-ATR spectra of the MOF-808(Zr) and MOF-808(Hf). (**c**) Powder X-ray diffraction patterns of simulated and experimental MOF-808 materials; the simulated diffractogram of MOF-808 structures were obtained from their crystallographic data deposited in the Cambridge Structural Database [29]. (**d**) Argon adsorption–desorption isotherms of the prepared MOF-808(Zr) and MOF-808(Zr). (**e**) EDX spectra and SEM images for the two experimental MOF materials.

The two porous MOF materials, MOF-808(Zr) and MOF-808(Hf), were prepared using solvothermal methods adapted from procedures previously reported. The purified materials were analyzed by FTIR-ATR (Figure 1b), powder XRD (Figure 1c), Argon adsorption–desorption isotherms (Figure 1d) and SEM/EDS (Figure 1e), confirming the preparation of the expected solid-state pure phases of both porous MOFs, MOF-808(Zr) and MOF-808(Hf). The typical extended crystalline structure of the MOF-808 family is shown in Figure 1a.

The FTIR-ATR spectra of Zr- and Hf-based MOFs reveal the characteristics absorption band expected from the MOFs framework in the 2000–400 cm^−1^ wavenumber region: a medium intensity band related to the bond vibrations of acetate groups coordinated with the oxo-clusters around 1650 cm^−1^; medium and strong bands around 1590 and 1390 cm^−1^ assigned to vibrational modes of the carboxylate groups, a medium absorption band at c.a. 1450 ascribed to aromatic (C=C) vibrational modes, a group of absorption bands associated with M−(μ_3_-O) framework bonds in the range 775–600 cm^−1^, and a weak band around 450 cm^−1^ assigned to M−(OC) bonds[40]. Powder XRD patterns of the isolated materials show the expected reflections of the MOF-808 crystalline phase, both in location and relative intensities [28], also neglecting the existence of any secondary crystalline phases when comparing with the suggested pattern from the crystallographic data (Figure 1c). The experimental diffractograms unequivocally confirm the preparation of the two MOF-808 materials with the desired crystalline phase, also pointing to a lower crystallinity of the MOF-808(Hf) relative to the Zr-based MOF. This fact is further corroborated by the SEM images of the two materials, which show larger and more regular particles for the MOF-808(Zr). The porosity of the material was analyzed by argon adsorption at −186 °C (Figure 1d). The type I adsorption/desorption isotherm exposed a permanent microporosity with a BET-specific surface area of around 910 m^2^/g (pore volume: 0.71 cm^3^/g at P/Po = 0.94739) and 998 m^2^/g (pore volume: 0.57 cm^3^/g at P/Po = 0.95708) for the MOF-808(Zr) and MOF-808(Hf), respectively. This difference was already verified and reported and attributed to the higher density of the Hf-based MOF material [41]. Combining the information given by the several characterization techniques performed, it is possible to confirm a successful preparation of porous MOF materials.

### 3.2. Acidity Characterization

The amount of acid sites existent in the two prepared MOF-808 structures was evaluated by measurement of the pH values of the solutions containing the Zr- and Hf-based MOF-808 materials solid dispersions, and the obtained results are presented in Table 1. These values suggested that MOF-808(Hf) contains stronger acid sites compared to MOF-808(Zr). Further, the concentration of released H^+^ ions was determined to be 0.75 and 0.43 mmol g^−1^ for MOF-808(Hf) and MOF-808(Zr), respectively, by the acid-base titrations.

### 3.3. Evaluation of Catalytic Activity

**Figure 2:**
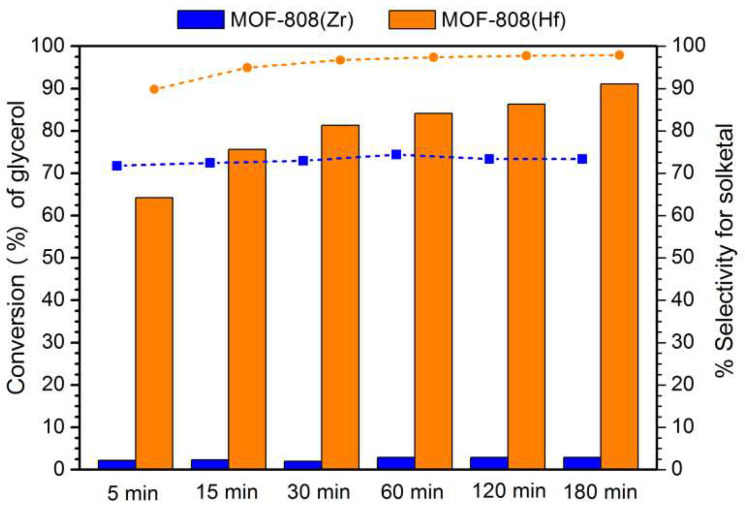
Conversion of glycerol by acetalization reaction catalyzed by MOF-808(Zr) and MOF-808(Hf) materials (15 mg) using a ratio of 1:6 glycerol/acetone and a temperature of 60 °C. In blue and orange, the conversion and selectivity data using MOF-808(Zr) and MOF-808(Hf) catalysts, respectively. Bars present conversion, and dots present solketal selectivity.

The two MOF-808 materials were used as heterogeneous catalysts in the valorization of glycerol with acetone to produce solketal. The catalytic activity was assessed at selected reaction conditions according to our previous work [42], i.e., temperature 333 K, molar ratio 1:6 glycerol/acetone, 4 wt% of catalyst relative to glycerol mass, and the absence of solvent. Figure 2 shows the catalytic performance of materials in terms of the overall glycerol conversion (in bars) and selectivity for solketal (dashed line). The MOF-808(Zr) catalyst showed much lower efficiency in glycerol conversion than the MOF-808(Hf) from the first minutes of the reaction. In fact, after 5 min, the Hf-based solid catalyst revealed more than 60% conversion, while the Zr-based catalyst presented less than 5%. The main reason that can explain the higher catalytic activity of the MOF-808(Hf) compared to the parent MOF-808(Zr) is probably the higher acidity of the Hf material when the same mass amount of the two catalysts is utilized. After 3 h of reaction, 91% of glycerol was converted using MOF-808(Hf) instead of 6% obtained with MOF-808(Zr) under the same reaction conditions. Even more important, the selectivity to solketal was also of 98% with only a vestigial amount of acetal (1,3-dioxane-5-methanol), while the selectivity with the Zr-based MOF was c.a. 75%.

### 3.4. Optimization of Acetalization Reaction

**Figure 3:**
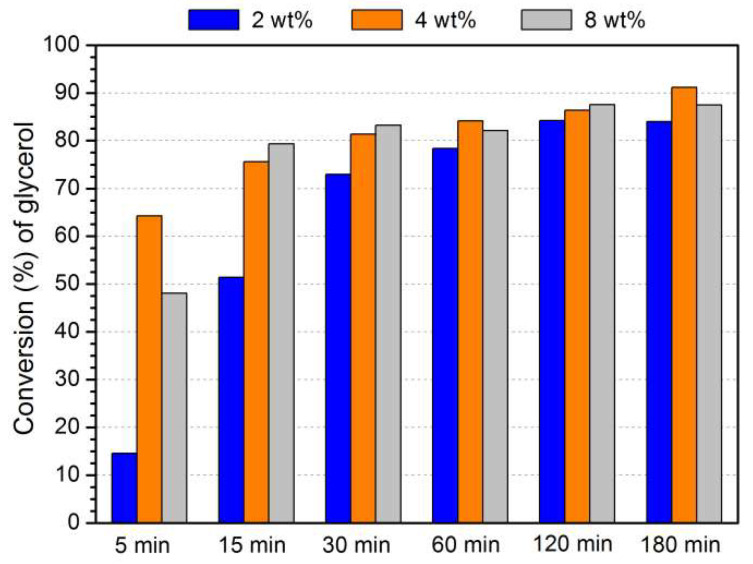
Conversion of glycerol catalyzed by different amounts of MOF-808(Hf) (2, 4, and 8 wt% from glycerol weight), using a 1:6 glycerol/acetone ratio and a temperature of 60 °C.

The material MOF-808(Hf) revealed higher catalytic efficiency than MOF-808(Zr) in the initial reaction experiments. Consequently, the optimization study was performed using the Hf-based material. Three different reaction parameters were investigated in the optimization process: the amount of catalyst (relative to glycerol mass), the ratio of glycerol/acetone, and the temperature. The initial conditions adopted were 15 mg of catalyst (4 wt% to glycerol), 1:6 glycerol/acetone ratio, and 60 °C. The effect of catalyst amount (7.5 mg, 2 wt% to glycerol; 15 mg, 4 wt% to glycerol; and 30 mg, 8 wt% to glycerol) in the acetalization reaction was analyzed, and the results are exhibited in Figure 3. The acetalization reaction profile is similar using the three different amounts of the catalyst after 2 h of reaction, mainly between 4 and 8 wt%. Using 8 wt% of catalyst, an excess of acidic centers is probably present, which did not contribute to the increased catalyst activity. Therefore, the catalyst amount used in the next studies was only 15 mg of MOF-808(Hf) (4 wt% to glycerol).

**Figure 4:**
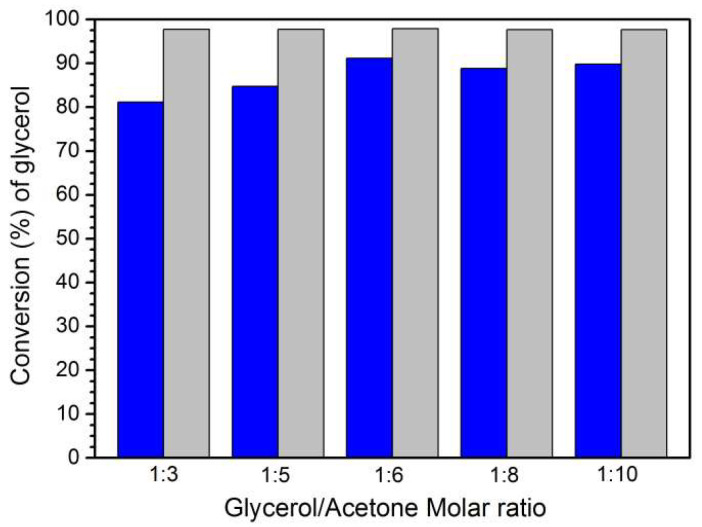
Conversion data obtained after 3 h of glycerol acetalization reaction catalyzed by MOF-808(Hf) using different ratio glycerol/acetone at 333 K (blue bars) and the corresponding solketal selectivity data (grey bars).

The acetalization of glycerol is a reversible process. Consequently, the utilization of an excess of acetone relative to glycerol will swing the equilibrium to the formation of a higher number of product(s) [22,43]. To assess this effect, the ratio of glycerol to acetone was varied: 1:3, 1:5, 1:6, 1:8, and 1:10 (Figure 4), maintaining the remaining reaction parameters invariable (15 mg of MOF-808(Hf) catalyst and 60 °C). After 3 h of acetalization reaction, it was verified that the increase in acetone amount from a ratio 1:3 to 1:6 favored higher glycerol conversion. Nevertheless, a superior increase in the amount of acetone (1:8 or 1:10) did not promote a higher conversion of glycerol. This probably happens due to the lower concentration of reactants since their probability to interact with the active site is decreased upon dilution [22]. Remarkably, the solketal selectivity was 98% for all glycerol/acetone ratios studied after 3 h of reaction.

**Figure 5:**
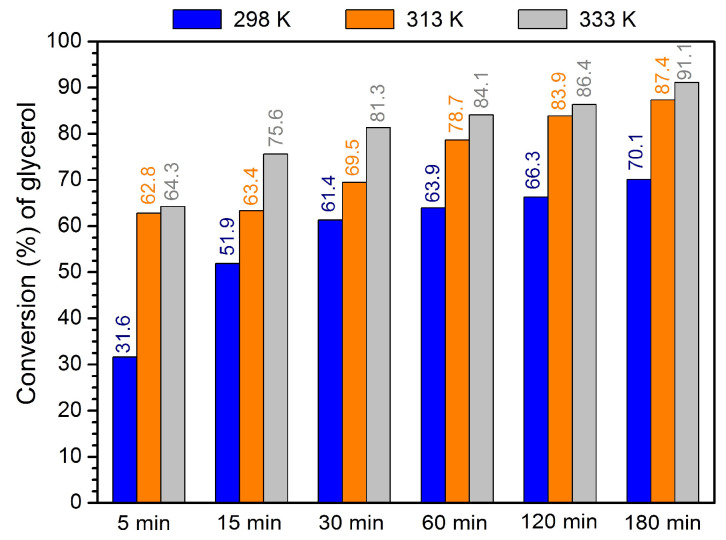
Conversion of glycerol by acetalization reaction using 1:6 ratio glycerol/acetone, catalyzed by 4 wt% of MOF-808(Hf), using different temperatures (298, 313, and 333 K).

The effect of temperature (298, 313, and 333 K) in the efficiency for glycerol acetalization conversion was analyzed using a 1:6 ratio of glycerol/acetone and 4 wt% (to glycerol mass) of MOF-808-H. The results depicted in Figure 5 reveal that an increase in the reaction temperature from 298 to 313 and 333 K led to an improvement in glycerol conversion after 3 h. After the first hour, the conversion of glycerol is similar, performing the reaction at 313 and 333 K, but still slightly higher using 333 K with 91% of conversion. Interestingly, the selectivity to solketal increases considerably with the reaction temperature: 58% using 298 K, 83% using 313 K, and 89% using 333 K, after 3 h of reaction. Higher temperature probably promotes a higher miscibility of the reaction medium and a higher contact between reactants. According to the results obtained for solketal (conversion and selectivity), the temperature selected for future experiments was 333 K.

### 3.5. Reutilization of MOF-808(Hf) Catalyst

**Figure 6:**
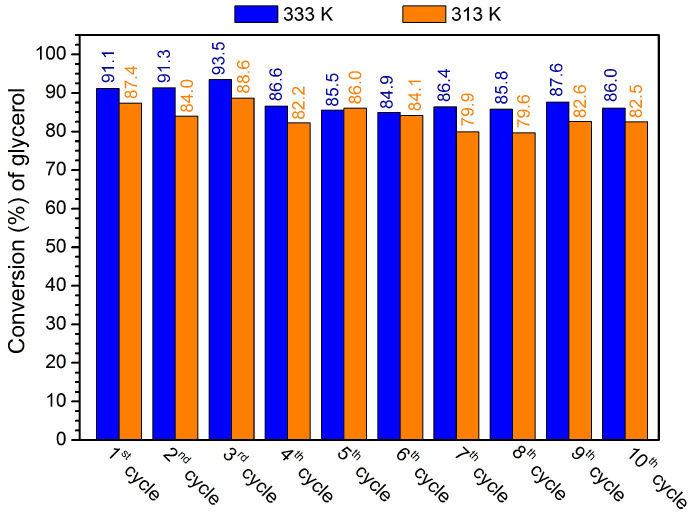
Conversion of glycerol by acetalization reaction performed for ten consecutive reactions, reusing the solid MOF-808(Hf) (4 wt%) catalyst (data obtained after 3 h). A ratio of 1:6 glycerol/acetone was used, and reaction cycles were performed at 313 and 333 K.

The capacity of reusing the MOF-808(Hf) as a catalyst was studied for the glycerol acetalization reaction using the optimized reaction parameters—15 mg of catalyst (containing 4 wt% compared to glycerol), 1:6 glycerol/acetone ratio—for ten consecutive cycles. In the reused procedure, after the first catalytic cycle, the reaction solution (containing products, acetone, and vestigial amount of glycerol) was removed, the solid was isolated, and without any treatment or washing process, a new portion of acetone and glycerol was added to run a consecutive reaction cycle. Figure 6 exhibits the results obtained for ten consecutive reusing cycles that were performed at 313 and 333 K. Comparing the reactions performed at these two temperatures, it is possible to observe that similar behavior was observed during the ten consecutive reaction cycles. The efficiency of the catalyst was practically maintained, and only a slight decrease in glycerol conversion could be observed after the 4th cycle. Most probably, this may be due to the occurrence of some catalyst mass loser when a high number of aliquots were taken during the 10 consecutive reactions.

The catalytic mechanism of the preparation of solketal from the acetalization of glycerol is well established and described in the literature, initially involving the formation of glycerol–acetone adduct. This last is transformed into a tertiary alcohol that interacts with the active acid sites present in MOF-808, to form a carbocation upon hydration. The hydroxyl groups of glycerol attack this carbocation to generate five and six membered products has recently been reported; the presence of defective linkers in the MOF framework can facilitate the transition from decarbonylation to the hydrodeoxygenation reaction [14,44].

### 3.6. Comparison with Reported Catalysts

The application of porous MOFs as heterogeneous catalysts for the acetalization of glycerol with acetone is very scarce, and the results already reported are summarized in Table 2. The first work was published by Timofeeva et al. in 2017, where it was reported that vanadium-based MOFs of type MIL-100 and MIL-47 showed high catalytic activity, achieving conversion of 83% after 1.5 h at 298 K [21]. However, the use of a high amount of acetonitrile as a solvent was a weak point of this work. Compared to the work presented here, no solvent was needed to find higher catalytic performance with MOF-808-Hf. Two years later, Santos-Vieira used a lanthanide Ln^3+^/Eu^3+^-based coordination polymer material to catalyze the same reaction. Identical catalytic efficiency was found using this material as the previous found by Timofeeva et al. work (84% conversion and 96% selectivity for solketal). However, Santos-Vieira’s group used a more expensive material, using higher temperature reaction and longer reaction time [23]. In the same year, Bakuru et al. used a family of UiO-66 materials as catalysts and, in this case, slightly higher conversion of glycerol (94%) with high selectivity for the solketal (97%) when the hafnium material (UiO-66-Hf) was used [22]. The order of catalytic activity between Hf- and Zr-based UiO-66 MOFs (UiO-66-Hf > UiO-66-Zr) is identical as obtained in the present work using MOF-808 material, and this is mainly due to the higher acidity of Hf MOF materials than the Zr. For higher acidity, higher glycerol conversions were obtained without harming the high selectivity of the desired product solketal. The efficiency and selectivity obtained with UiO-66(Hf) reported by Bakuru et al. are identical to those obtained with the MOF-808(Hf) in this work [22]. Nevertheless, it is important to note that a much higher amount of catalyst was used (10 wt% of total glycerol used) than the present work (4 wt%). On the other hand, recycling studies performed with UiO-66(Hf) revealed some loss of activity, justified by the occurrence of pores blockage promoted by reactants and products trapped in the pores of UiO-66(Hf). The pore diameter and the window openings of UiO-66 are smaller (12 and 7 Å, respectively) than the same for MOF-808 (18 and 14 Å, respectively), decreasing the possibility of pore blockage and the unfeasibility of active center access, using the MOF-808(Hf) material.

### 3.7. Catalyst Stability

**Figure 7:**
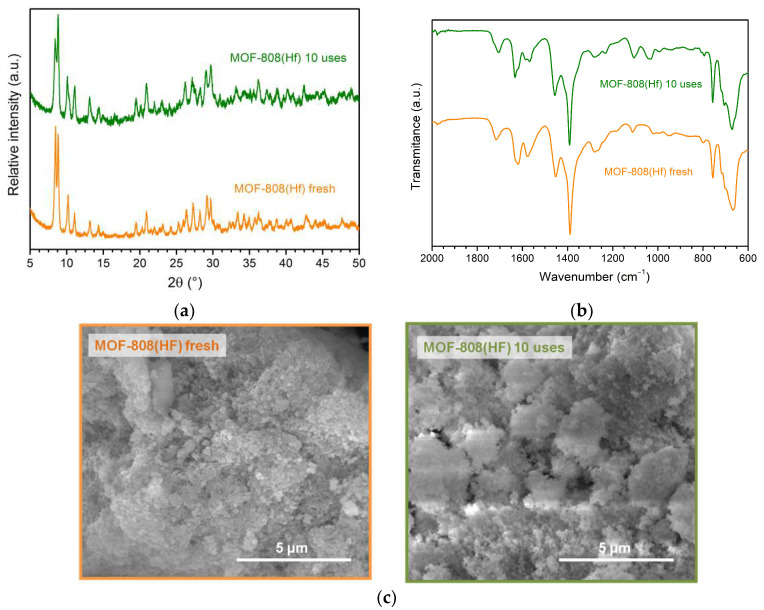
(**a**) X-ray diffraction patterns, (**b**) FITR-ATR spectra, and (**c**) SEM images of fresh and used MOF-808(Hf) catalyst after ten consecutive reactions.

The powder XRD, vibrational spectroscopy, and SEM images of the recovered catalyst after ten cycles were carried out to evaluate the MOF-808(Hf) structure stability after their catalytic application. The powder XRD pattern of the recovered catalyst after ten cycles evidenced the same crystalline structure of pristine MOF-808(Hf). Moreover, the recovery of the solid catalyst was practically complete after each cycle. In addition, the reused catalyst was also characterized by FTIR-ATR and SEM (Figure 7). This complementary characterization confirms that the structure of the Hf-based MOF material remains unchanged after reuse, showing a vibrational spectrum similar to the fresh material, and no significant morphological changes are observed. All these results demonstrated that the MOF-808(Hf) material is a catalytically active material, easy to recover, and reusable in the revalorization of glycerol.

## 4. Concluding Remarks

MOF-808 structures containing Zr and Hf, MOF-808(Zr) and MOF-808(Hf), were successfully prepared and used as heterogeneous catalysts for the reaction of acetalization of glycerol with acetone for the first time. The MOF-808(Hf) showed to be much more active than the MOF-808(Zr), and its high efficiency was attributed to the higher number of acid centers present in the Hf-based porous material when the same amount of material was used. After 5 min of reaction, nearly 60% of glycerol was converted to solketal instead of less than 5% using the Zr-based material. After 3 h of reaction, 91% of glycerol was converted using MOF-808(Hf) instead of 6% obtained with MOF-808(Zr) under the same reaction conditions. These results were obtained after the optimization of various parameters, such as temperature (333 K), catalyst amount (4 wt% of the total glycerol used), and ratio glycerol/acetone (1:6). The solid catalyst was then reused for ten consecutive reaction cycles without washing or treatment between cycles. Its catalytic activity was maintained at 333 K, and its structural stability was confirmed after the ten cycles by FTIR, XRD, and SEM. In fact, the straightforward prepared MOF-808(Hf) revealed potential at the laboratory scale to be applied as a sustainable catalyst in this valorization process of glycerol residue. Consequently, these promising results deserve future scale-up investigation of the reported system.

## Figures and Tables

**Table 1:** pH values and acidity of the MOF-808 materials.

MOF	pH (Before Titration)	Acidity (mmol g^−1^)
MOF-808(Hf)	3.61	0.7505
MOF-808(Zr)	4.35	0.4292

**Table 2:** pH values and acidity of the MOFs-808 materials.

Catalyst	Temperature (K)	Glycerol/Acetone	Time (h)	Conversion (%)	Reference
**MIL-100(V)**	298	1:5	1.5	83 (98)	[21]
**MIL-47(V)**	298	1:5	1.5	73 (87)	[21]
**UiO-66-Zr**	r.t.	1:4	1	1.5 (73)	[22]
**UiO-66-Hf**	r.t.	1:4	1	94 (97)	[22]
**UiO-66-SO_3_H**	333	1:10	1	60 (99)	[24]
**Mil-118-SnO_2_**	reflux	1:10	4	76 (97)	[25]
**UAV-63**	328	1:10	6	84(96)	[23]
**MOF-808(Zr)**	333	1:6	3	6 (100)	This work
**MOF-808(Hf)**	333	1:6	3	91 (98)	This work

r.t., room temperature.

## Data Availability

Not applicable.
